# “He was the one with the gun!” Associative memory for white and black faces seen with weapons

**DOI:** 10.1186/s41235-022-00355-z

**Published:** 2022-01-31

**Authors:** William Blake Erickson, Arianna Wright, Moshe Naveh-Benjamin

**Affiliations:** 1 Department of Life Sciences, Texas A&M University – San Antonio, One University Way, San Antonio, TX 78224 USA; 2 Department of Psychology, University of Wisconsin – Madison, Madison, USA; 3 Department of Psychological Sciences, University of Missouri, Columbia, USA

## Abstract

Much research has found that implicit associations between Black male faces and aggression affect dispositional judgments and decision-making, but there have been few investigations into downstream effects on explicit episodic memory. The current experiment tested whether such implicit associations interact with explicit recognition memory using an associative memory paradigm in younger and older adults. Participants studied image pairs featuring faces (of Black or White males) alongside handheld objects (uncategorized, kitchenware, or weapons) and later were tested on their recognition memory for faces, objects, and face/object pairings. Younger adults were further divided into full and divided attention encoding groups. All participants then took the race faces implicit association test. Memory for image pairs was poorer than memory for individual face or object images, particularly among older adults, extending the empirical support for the age-related associative memory deficit hypothesis (Naveh-Benjamin in J Exp Psychol Learn Mem Cognit 26:1170–1187, 2000) to associations between racial faces and objects. Our primary hypothesis—that older adults’ associative memory deficit would be reduced under Black/weapon pairings due to their being schematically related stimuli—was not confirmed. Younger adults and especially older ones, who were predominantly White, exhibited an own-race recognition bias. In addition, older adults showed more negative implicit bias toward Black faces. Importantly, mixed linear analyses revealed that negative implicit associations for Black faces predicted increased explicit associative memory false alarm rates among older adults. Such a pattern may have implications for the criminal justice system, particularly when weighting eyewitness testimony from older adults.

## Significance statement

Highly publicized killings of unarmed Black Americans by police officers have drawn the attention of academics from many fields united in the search for causes and solutions to this social malady. Much psychophysical and social-cognitive investigations into the roles of implicit bias, stereotypes, and intergroup exposure on shaping attitudes toward Black men as innately aggressive were conducted. In real cases, this has extended to misidentification of handheld objects (e.g., candy bars, cellphones) as weapons. The current experiment extended these findings to human memory and aging. Younger and older participants studied sets of image pairs featuring faces alongside handheld objects. Faces were Black or White males, and objects were schematically uncategorized (i.e., belonging to no specific, coherent category), kitchenware, or weapons. Recognition memory tests followed for faces, objects, and face/object pairs. This latter test was of central interest, consisting of faces displayed alongside objects as during study, but half of these faces were recombined with objects they were not seen with during study. This provided a test of whether participants would mistake faces as having been seen alongside a different type of object than they were seen with initially. Importantly, we found that older adults—particularly those with an unfavorable bias against Black individuals (as measured by the Implicit Association Test-IAT)—more often indicate during these tests that Black faces were paired with different objects, including weapons than they do so for White faces, potentially due to an overreliance on automatic decision strategies rooted in social schemas. Such mistaken identity related to face and object associations may have myriad downstream effects on criminal justice outcomes for Black Americans affected by faulty eyewitness testimony.

## Introduction

Learning and applying object category classifications is vital to everyday perception and decision-making, and successful categorical learning is therefore a hallmark of cognitive development (e.g., Piaget, 1954). In this matter, faces are like any other object, and they too come in a myriad of categories. For example, faces may be categorized by characteristics of sex, culture-specific features, or familiarity. Indeed, when encountering unfamiliar faces, the human visual processing system first categorizes the new face before attending to and encoding individuating features (Hugenberg et al., 2010). Facial race and ethnicity are powerful categories that are encoded immediately by individuals encountering unfamiliar faces. Although not problematic in itself, facial categorization can prime stereotyping for an individual based on their group membership, producing negative judgments without direct evidence (Wilson et al., 2017). The study reported here examines whether race-based facial stereotypes influence associative memory when faces are paired with various categories of objects during encoding—namely, objects congruous or incongruous to the facial stereotype. In addition, we were interested in determining whether stereotypic congruity reduces the general deficit in associative memory observed in older adults (aged 65+) relative to their younger counterparts (Naveh-Benjamin, 2000).

### Black faces and aggression

The current study was directly inspired by the increasing media attention to police-involved shootings of unarmed Black Americans. In an important subset of these cases, officers have misidentified handheld objects as weapons; brandishing the objects out of fear and resulting in shooting their weapons for their own lives as well as the lives of bystanders. For example, one case reported police firing 137 bullets into a car after misidentifying a slice of pizza the passenger was eating as a gun (“6 Cleveland cops fired over, 2012 chase,” 2016). The driver and passenger, both African American, were consequently killed. To note, cases like this vary widely in their specific details. These include situational reasons why the police may misidentify the object,^1^ whether the victim is or resembles a suspect in a violent crime, the officer holds some form of bias and whether the police followed standard protocol at local, state, and federal levels regarding use of force.

One possible mechanism that has received much attention involves the association of Black individuals, particularly Black men, with aggression and violence more often, compared to other ethnic groups. In an early study, Sagar and Schofield (1980) asked preadolescents to rate Black and White individuals’ behaviors in scenarios featuring ambiguous interactions. Black individuals’ behaviors were more often interpreted as more aggressive than the same behaviors enacted by White actors. This effect was observed even in scenarios involving no physical contact, which the authors attributed to reliance on social stereotypes. Hugenberg and Bodenhausen (2003) demonstrated that implicit (cf., explicit) bias predicts a greater tendency to prematurely judge onset of expressions of anger in Black faces compared to White faces. Recently, Wilson et al. (2017) asked participants to rate images of Black men’s bodies, varying the images along several measures such as height, weight, and muscle mass. Across several studies, the authors found that Black males are perceived larger and more threatening than images of White males with matching biometrics.

Other studies have directly applied categorical associations to investigations of “shooter bias” scenarios. In such studies, participants are presented with simulations of everyday scenes overlaid with individuals of various races holding weapons or other objects. Participants are then told to press one button to “shoot” at those holding weapons and press another button signifying “don’t shoot” at those holding other objects. Correll et al. (2002) found in such a paradigm that within an 850 ms window, participants made few errors regardless of target race. However, reaction times to shoot at Black actors holding weapons were significantly shorter than for White actors holding weapons, and “don’t shoot” reaction times were shorter for unarmed White actors compared to unarmed Black actors. In a second study, the authors implemented a shorter response window of 630 ms, which yielded unarmed Black actors receiving double the errors (i.e., “shoot” decisions) compared to when they were armed. White errors were not different between armed and unarmed individuals. A third study replicated the first but included surveys of stereotype endorsement and prejudiced beliefs. It revealed that mere knowledge of cultural stereotypes positively correlated with “shooter bias,” and endorsement of these stereotypes was not necessary. The authors concluded with a preliminary model of the shooter bias phenomenon invoking fast, automatic association of ethnicity and aggression for Black individuals which reduces the number of relevant features required to conclude that a handheld object is a weapon. People are then more likely to make a “shoot” decision, which increases errors. Follow-up studies found that exposure to media stories about crimes committed by Black individuals and increasing prevalence of armed Black individuals within the simulations exacerbated shooter bias by confirming the stereotype and increasing the strength of the association with aggression (Correll et al., 2007a, 2007b). Elsewhere, the shooter bias has been found for West Asian men in European countries with large migrant populations (Essien et al., 2017; Fleming et al., 2010).^2^

Schematic association between Black faces and weapons has yielded effects in other relevant decision-making outcomes as well. For example, exposure to Black faces prior to presenting images of objects increases accuracy at object categorization judgments when the subsequent object is a weapon compared to a non-weapon object, whereas this association is weaker when weapons are preceded by White faces (see Payne, 2006, for a review). More recently, investigation in eyewitness memory has found that the so-called *weapon focus effect*, wherein post-event identification of criminal perpetrators holding weapons is less accurate than identifications when a weapon is not present during the crime (e.g., Erickson et al., 2014), is ameliorated when the perpetrator is an Black male, and more so when he wears stereotypic “hip-hop” clothing (Pickel & Sneyd, 2017). Taken together, these findings present a stable, replicable tendency to associate male Black faces with aggression. This association then biases object identification judgments and affects later facial recognition.

Surprisingly, a relationship between this bias and conscious endorsement of racist attitudes is not required, and even Black subjects exhibit bias in the same direction as White subjects (e.g., Correll et al., 2002). Researchers have speculated that mere knowledge of cultural stereotypes is enough to retain the associative bond (Arkes & Tetlock, 2004; Correll et al., 2002). The current study extends the effect of this association to another domain: the age-related associative memory deficit observed in older adult populations. Importantly, we investigate whether the schematic relationship described thus far is strong enough to ameliorate the associative memory deficit within older adults. Before outlining the method of the current study, we review the relevant literature of age-related memory declines.

### Age-related memory declines

Older adults have difficulty retaining episodic information, which requires the cognitive resources to encode events and their specific corresponding contextual details (Naveh-Benjamin & Old, 2008; Zacks et al., 2000). In such events, the goal is to retain rich contextual details of single items during the encoding process to form a smooth, cohesive episodic memory, which should be reminiscent of their individual component elements as well as inter-component associations (Tulving et al., 1983). Such episodic memory involves a collection of many single unit items of information, including emotions, timing, and contextual details of the experienced event.

One of the effects of normal adult aging on cognition is a reduction in associative memory accuracy. Specifically, older adults more often than younger adults fail to bind individual components together to form cohesive episodes in what Naveh-Benjamin (2000) refers to as the “associative deficit hypothesis” (ADH). In his initial study, the author hypothesized that the deficit in older adults’ episodic memory may result from a declined ability to create links when binding together single units of information. Participants were asked to study lists of word-nonword pairs in preparation for item and associative memory tests which would follow. There were three tests: word recognition, nonword recognition, and association recognition. Word and nonword tests contained equal numbers of targets and distractors, whereas the association test featured half intact pairs and half recombined pairs, such that all the components in the associative tests were previously studied. Results indicated that the older adults were less accurate than younger adults in the associative memory tests, but this difference was less pronounced in the word and the nonword tests. The ADH has since been replicated in many studies using a variety of stimuli, encoding, and retrieval conditions (see a meta-analysis by Old & Naveh-Benjamin, 2008).

Most relevant to the current study is follow-up research finding that the ADH can be ameliorated when component stimuli are categorically related (Naveh-Benjamin, 2000, Experiment 4; Naveh-Benjamin et al., 2003; Mohanty et al., 2016). This better linking of single units together is achieved because people can rely on preexisting knowledge about associations between components rather than creating their own associations at encoding. This in turn requires fewer cognitive resources to be devoted to creating new connections during the encoding of the event, thus allowing a better encoding of the bindings between the components. In the previous section, we outlined how several simple decision-making tasks can be affected by categorical associations involving face stimuli, namely the face’s race. This association has downstream effects on memory as well. Ackerman et al. (2006), for example, found that participants were better at remembering Black faces when they expressed faces of anger during encoding versus when they expressed neutrally. This provides evidence that associative memory for faces and their expressions might be improved when components already have a meaningful link. However, this could also lead to false alarm errors when a previously unstudied Black face displaying a neutral expression is tested with an angry expression. Such faces would be categorically congruent but nonetheless incorrectly recognized.

Declining memory that accompanies advanced age is also relevant in many day-to-day situations. For example, improperly binding component stimuli may disrupt memory for witnessed crimes (e.g., who did what, or who has held the weapon) for which older adults exhibit less accurate memory than younger adults (Erickson et al., 2016). However, facial memory may be enhanced if the faces are categorically congruent with the event. Eyewitness simulation research comparing older and younger adults’ memories have not systematically examined these relevant variables, but it may be that facial memory would be enhanced if faces are paired with categorically congruent objects. Above we outlined research finding that Black faces are more likely to be associated with aggression and threat than White faces (e.g., Hugenberg & Bodenhausen, 2003). This stereotypic association may yield more accurate recognition of face-object pairs when component stimuli that are semantically related—namely, when a Black face is paired with a weapon. The current study examines these issues, integrating findings from cognitive, social, and eyewitness research to determine if some face-object pairings are easier to recognize later because they are semantically related, and whether this could help older adults’ associative memory deficit.

### Measuring implicit associations

Semantic and categorical links can produce conscious, explicit associations and unconscious, implicit associations. Almost all of these associations are benign, such as the conceptual links between two synonyms or immediate disgust at the sight of food one dislikes. However, in these and most cases, people will freely admit to their explicit attitudes. As overt racist attitudes have become socially unacceptable in contemporary culture, people are unlikely to openly admit holding such views due to social desirability bias even in the context of an experiment where anonymity is guaranteed. Moreover, as mentioned above, such implicit associations may nonetheless present in people who do not share such explicit views, and these may influence cognition and consequent behaviors. The prevailing tool to measure implicit racial bias is the Implicit Association Test (IAT; Greenwald et al., 1998). The IAT is a psychophysical task which has participants categorize faces as White or Black and words as “Good” or “Bad” as quickly as possible. In a computerized interface, participants categorize these stimuli using a key from the left side of the keyboard for White and “Good” words and a different key from the right side of the keyboard for Black and “Bad” words. After two blocks of this configuration, the category keys for White and Black faces swap, but the word category keys remain unchanged. If participants are slower or faster to categorize faces after this reconfiguration occurs, this is treated as evidence that the participant harbors an implicit attitude about the face's race valance toward whatever word type was categorized on the side of that face race. Although there has been criticism of the IAT's validity and reliability as a tool for predicting overt attitudes and discriminatory behavior (e.g., Gawronski, 2019), it may prove useful in the current study as a means to verify whether participants' personal schemas for race relate to associative memory within the brief timeframe of a single experiment session. Namely, IAT performance may predict associative memory accuracy for Black faces paired with weapons, both of which may elicit negative emotions.

Still another way to determine if underlying semantic associations predict memory accuracy is to ask participants to study face/object pairs while carrying out a different ongoing task. Memory accuracy is sensitive to such divided attention at encoding but not retrieval (Craik et al., 1996). In turn, individuals studying under divided attention rely more on quick, automatic processing based on preexisting associations such as those that may be shared between Black male faces and weapons. Previous research using an associative memory paradigm has found that younger adults studying stimuli under divided attention perform poorer overall at recognition memory tasks than younger adults studying under full attention, but they do not exhibit an associative memory deficit as older adults do (Naveh-Benjamin et al., 2003). We include a manipulation of attention by having a group of younger adults undergo a concurrent task during encoding to replicate these results as well as determine whether they extend to the domain of face race/object associations.

### The present study

The current study integrates elements from all the research described thus far. Primarily, it examines whether threatening objects are more likely to be associated in memory with Black faces than with White faces and whether this is the case in both younger and older adults as well as in younger adults under divided attention at encoding. The use of younger adults under divided attention condition may allow us to assess whether such enhanced bindings of faces and threatening objects may happen somewhat automatically, under depleted attentional resources, when part of the participants’ attention is devoted to a concurrent task. Participants viewed series of face-object pairs and were explicitly told to remember them for later face, object, and face-object associative memory tests. Half of the faces were Black and half were White, and each face type was paired with equal frequency with weapons, kitchenware (which represent a coherent category of related objects), and schematically uncategorical objects belonging to neither weapon nor kitchenware categories. In addition, we administered the Race Faces variant of the implicit association test to conduct analyses investigating the relationship between participants’ implicit associations of race-based faces with their associative memories. We put forth the following hypotheses:

- Relative to younger adults under full attention, older adults will show an associative memory deficit: Their memory for the associations between the components of the episodes will be poorer relative to their memory of the components themselves.
- Both younger and older adults will exhibit better associative memory when Black faces are paired with weapons (rather than with other objects) due to their schematic association.
- Age-related associative memory deficits will decrease when Black faces are paired with weapons (rather than with other objects) due to the older adults’ generational cohort experiencing a lifetime of exposure to explicit cultural stereotypes about different races, strengthening the encoding of these associations.
- Younger adults studying stimuli under divided attention would perform relatively poorer than younger adults under full attention. However, given the association between Black faces and weapons, this subset of pairings will rely on automatic encoding resulting in more accurate memory even under divided attention, particularly among those who associate Black faces with negativity as measured by the implicit association test (see below).
- For the analyses of implicit bias’s relationship with associative memory performance, we predicted two potential outcomes: First, participants implicitly associating Black faces with negative words in the IAT may more often mistake Black faces that were not paired with weapons at study for being initially paired with weapons. In this scenario, such participants would commit more false alarms of Black face/weapon test pairs relative to other face/object combinations. Alternatively, participants implicitly associating Black faces with negative words may more accurately remember Black faces that were paired with weapons at study as these test pairs benefit from preexisting semantic associations that would strengthen component binding at study.

## Methods

### Participants

The sample included 68 younger adults and 43 older adults. Young adult participants were undergraduate students from the University of Missouri and recruited from the introductory psychology research pool. They ranged from 18 to 38 years of age (*M *= 19.61, *SD * = 2.58) and contained 36 women and 32 men. Among younger adults, 56 identified as “White,” five as “Black,” three as “Asian,” two as “Native American/Indian,” two as “Mixed,” and one declined to answer. These participants received completion credits for their participation in the study. Older adult participants were recruited from the laboratory’s own subject pool comprising residents from local communities of Central Missouri. All older participants were required to take part in a phone interview with one of the researchers to complete a general health questionnaire before participating. The older adults included for this study reported overall good health and did not have any medical conditions that could affect cognitive functioning. Older adults ranged from 54 to 89 years of age (*M* = 72.79, *SD * = 7.04) and included 34 women and nine men. Among older adults, 41 identified as “White,” 1 as “Black,” and 1 as “Asian.” They were compensated $15 for their participation. Because the IAT component was introduced soon after data collection began, 12 older adults and nine younger adults in the full attention group were not included in analyses featuring IAT scores.

### Design

The experiment employed a 3 (group: older adults, younger adults at full attention, younger adults under divided attention at study) × 3 (object type: weapon, kitchenware, uncategorical) × 2 (face type: Black and White) × 2 (memory test: item vs. associative) mixed factorial design. The within-subjects variables were face type, object type, and test type. The between-subjects variable was group, with younger adults randomly assigned to one of the attention conditions (Full Attention *N* = 39; Divided Attention *N* = 29) and older adults always studying under full attention. The dependent variable was memory accuracy for the item and associative memory tests. Hits, false alarms, and a measure of discriminability (proportion of hits minus proportion of false alarms) were used as measures for accuracy, and IAT scores were used as the measure of implicit racial bias.

The final sample size matches sizes recruited for previous associative deficit research (e.g., Naveh-Benjamin and Kilb (2014) sampled 30 younger and 31 older adults), which have proven robust for within-subjects factors with multiple observations per participant. Our oversampling of older adults and younger adults for the full attention group was done so that the final analyses of IAT scores were equitable across groups.

### Materials

Adult male face images were selected from the MORPH database (Ricanek & Tesafaye, 2006). MORPH includes images of over 13,000 identities and several ethnic groups, with many identities featuring multiple photographs at different ages. MORPH’s facial images were all taken in a controlled setting featuring the same backgrounds, luminosity, and visual angle that faces take up within the images. For the current study, the first and second authors chose 90 Black male and 90 White male faces, taking care to ensure each face was upright, posing a neutral expression, and visually distinctive from the others within each race. Similar-looking faces were then spread across different blocks of the experiment (see Procedure).

Forty-eight object images for each object type were chosen. Object images were taken from searching keywords on Google Images for guns, knives, kitchenware, and hand-held objects. Some of the uncategorical objects were chosen on the basis of being the same type of object having been reported in media coverage of police misidentifying objects as weapons. Unlike faces, objects vary much more widely in terms of their specific structural features (e.g., various handguns, rifles, and knives are designed rather differently), which allowed us to select an even more discriminable array objects for each of the three types used in this experiment.

Face and object images were paired such that an equal number of each of the six possible combinations of object types and race (e.g., White and weapon) appeared in six study lists of 20 image pairs. Eighteen pairs went on to be included in test events, and single buffer pairs that were never tested began and ended each study phase to control for primacy and recency effects. To increase discriminability, faces and objects were distributed among the lists to maximize perceptual differences (e.g., if two pistols appeared nearly identical, they were assigned to different blocks).

Each test block featured 12 test trials. Face and object tests featured equal numbers of each face or object type, half of which were targets from the study phase and half of which were distractors. Association tests featured faces and objects that were always previously studied, but half of the test pairs were intact from the study phase and the other half were recombined pairs. Importantly, face-object recombination trials occurred within each race. For example, a Black face that was presented with an uncategorical object during the study phase would be recombined with a weapon which was previously paired with another Black face. That face in turn would be recombined with a kitchenware object, and so on. This recombination scheme was chosen so that recombination trials could not be easily identified as such by participants because objects were recombined across face race. Targets and distractors, intact and recombined pairs, and test order were counterbalanced. In addition, face race was counterbalanced within object type.

Younger adults in the divided attention condition engaged in a secondary task during the study phases. The secondary task required participants to respond to a series of tones during either the study or the test phase. The tones were three easily discriminable frequencies: low, medium, and high. After hearing a tone, the participant responded by pressing the appropriate labeled keyboard button in accordance with frequency type, which triggered the next tone appearance. Participants first performed the tone response task by itself to serve as a baseline. This baseline was used to determine secondary task performance costs that had occurred during the study phases for the divided attention condition.

### Procedure

#### Explicit memory task

**Fig. 1:**
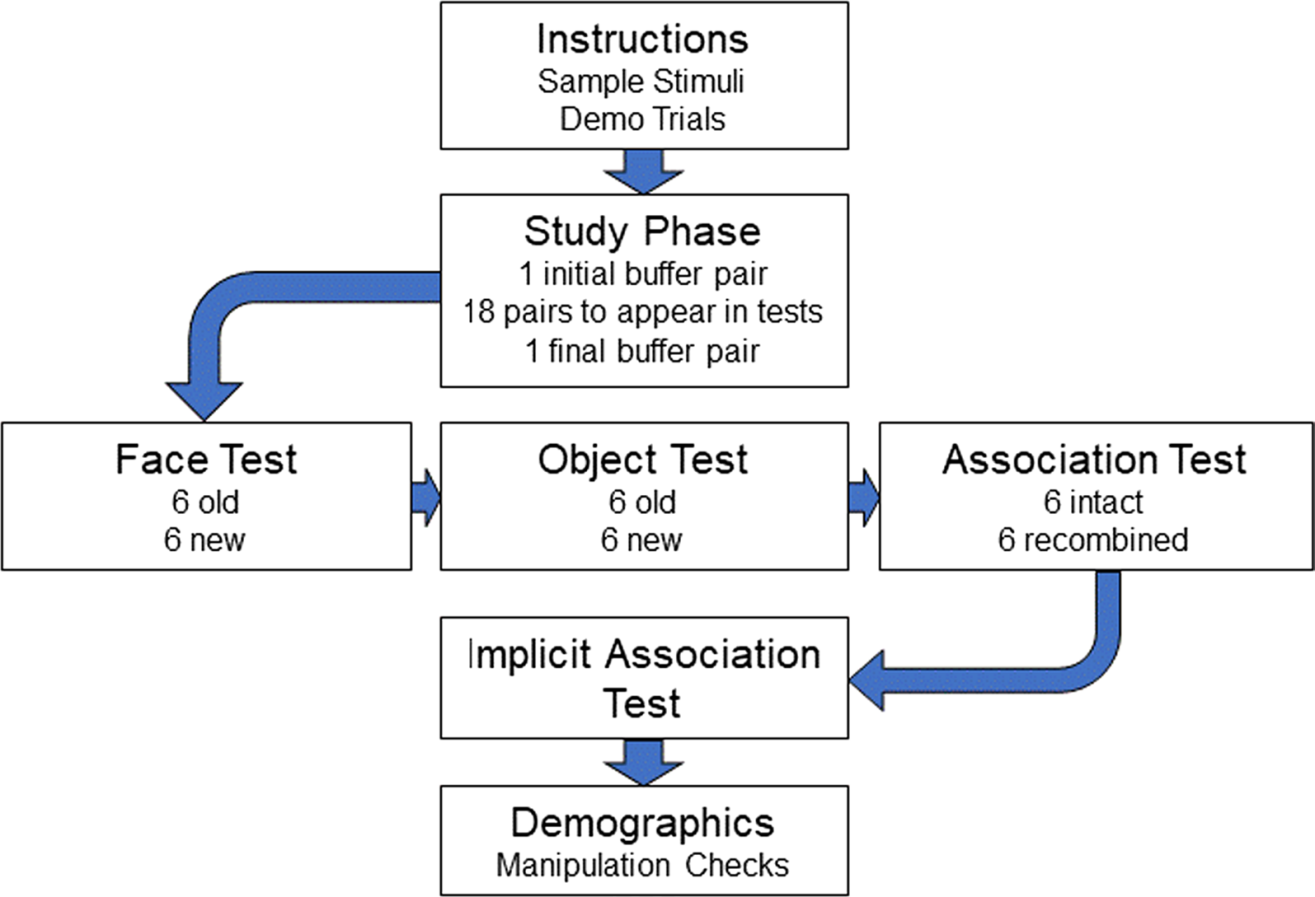
Schematic portraying general flow of events in each experimental session. Recognition data were collected in six experimental blocks featuring a study phase and the three test phases. Test phase order was counterbalanced among participants

Participants were tested individually in quiet laboratory rooms with an experimenter present. All stimuli and tests were presented and recorded on computers running E-Prime 2.0 (Psychology Software Tools, 2012). Figure 1 displays general session flow. After being presented with a consent form, the experimenter read onscreen instructions to participants and ensured that participants understood the nature of the experiment. After viewing examples of stimuli, participants underwent practice trials for study and test blocks. At study, image pairs displayed for seven seconds each. Face images always appeared on the left of the screen, and objects always appeared on the right. Tones playing during the secondary task for younger adults in the divided attention condition played for 500 ms, and a new tone played 300 ms after participants made a response. If participants failed to make a response, a new tone would play 2 s afterward. At test, trials had no time limits, and faces and objects appeared on the left and right of the screen, respectively. After practice, participants were administered the six main study-test blocks in a random order. E-Prime recorded test trial accuracy. The overall task took approximately 45 min to complete.

#### Implicit association task

After the conclusion of the abovementioned memory task, we set out to validate our interpretation of implicit associations by having participants complete the “Race faces” variant of the implicit association task (IAT) offered by Project Implicit (see Nosek et al., 2007, for a full description of the task and underlying assumptions). Although the face and word stimuli were sourced from Project Implicit, the task itself was rebuilt in E-Prime for ease of administration. Participants were first given basic instructions on the nature of the categorization procedure, specifically that they would categorize words and faces using the “E” and “I” keys on the keyboard as quickly as possible. They began with a version of the task featuring 20 trials featuring faces only. If participants saw a Black face, they were told to indicate so by pressing the “I” key and to categorize White faces by pressing the “E” key. The next block featured 20 trials of words only. If participants saw a “Bad” word (e.g., “Nasty,” “Terrible,” etc.), they would indicate so by pressing the “E” key and to categorize “Good” words (e.g., “Peace,” “Wonderful,” etc.) by pressing the “I” key. The next block randomly intermixed 10 words and 10 faces, although categorization keys remained the same as the previous blocks. The fourth block intermixed 20 words and 20 faces, again retaining the categorization keys. The fifth block presented 40 trials of faces only, but this time participants were instructed to press the “I” key for White faces and the “E” key for Black faces. A 20 trial practice block intermixing faces and words, with faces using the new keys, followed. The seventh and final block presented 40 trials intermixing faces and words, with faces using the new keys. The fourth and seventh blocks in this task are of primary experimental interest—if participants’ average response latencies were faster or slower after faces swap response keys, this indicates a positive or negative implicit bias for the race to which the face belongs.

#### Post-test questionnaires

After the IAT, participants completed questionnaires assessing demographic information as well as probing for their perceptions of the nature of the experiment and the difficulty of the tasks. After these were completed, participants were debriefed, and the experimenter answered any questions participants asked pertaining to the study. Compensation was then provided as prescribed for each age group.

## Results

Hits, false alarms, and a measure of discriminability (proportion of hits minus proportion of false alarms) were computed separately. A series of analyses on each outcome comparing older adults with younger adults at full attention, comparing older adults with younger adults at divided attention, and comparing younger adults at full attention with younger adults at divided attention were carried out. Discriminability represented our primary measure of memory accuracy (see Table 1 for means and standard deviations), whereas analyses of hits and false alarms allowed us to explore fine-grained effects on these types of decisions.

**Table 1:** Mean hit rates (H), false alarm rates (F), and memory discriminability (H–F) for each age group across each test stimulus type

Outcome	Faces		Objects			Associations					
	Black	White	Kit	Weap	Unc	B/Kit	B/Weap	B/Unc	W/Kit	W/Weap	W/Unc
*YA-FA*											
Hits	.73 (.17)	.73 (.17)	.83 (.15)	.84 (.13)	.85 (.17)	.72 (.24)	.79 (.22)	.87 (.17)	.76 (.21)	.73 (.21)	.85 (.16)
False Alarms	.19 (.16)	.12 (.11)	.07 (.10)	.17 (.16)	.02 (.04)	.33 (.20)	.30 (.22)	.33 (.23)	.32 (.25)	.29 (.24)	.25 (.19)
H–F	.54 (.24)	.61 (.21)	.75 (.18)	.67 (.20)	.83 (.17)	.39 (.34)	.49 (.32)	.54 (.32)	.44 (.31)	.44 (.34)	.61 (.29)
*YA-DA*											
Hits	.63 (.18)	.59 (.16)	.68 (.19)	.72 (.16)	.68 (.20)	.64 (.22)	.67 (.29)	.72 (.17)	.55 (.25)	.64 (.25)	.67 (.22)
False Alarms	.42 (.22)	.32 (.19)	.26 (.22)	.32 (.20)	.07 (.11)	.52 (.26)	.43 (.22)	.44 (.24)	.45 (.18)	.45 (.22)	.55 (.20)
H–F	.21 (.21)	.27 (.23)	.42 (.20)	.40 (.23)	.61 (.23)	.13 (.27)	.24 (.32)	.29 (.28)	.10 (.30)	18 (.31)	.12 (.27)
*Older*											
Hits	.84 (.12)	.84 (.15)	.88 (.12)	.89 (.10)	.93 (.08)	.75 (.18)	.76 (.16)	.86 (.14)	.72 (.20)	.73 (.22)	.83 (.15)
False Alarms	.31 (.21)	.15 (.13)	.08 (.10)	.28 (.17)	.03 (.06)	.37 (.23)	.33 (.26)	.39 (.27)	.40 (.26)	.31 (.25)	.34 (.24)
H–F	.52 (.24)	.68 (.19)	.79 (.14)	.61 (.19)	.89 (.11)	.38 (.28)	.43 (.26)	.47 (.26)	.31 (.29)	.43 (.29)	.48 (.27)

Standard deviations are presented in parentheses

YA, Young Adult; FA, Full Attention; DA, Divided Attention; Kit, Kitchenware; Weap, Weapon; Unc, Uncategorical

**Fig. 2:**
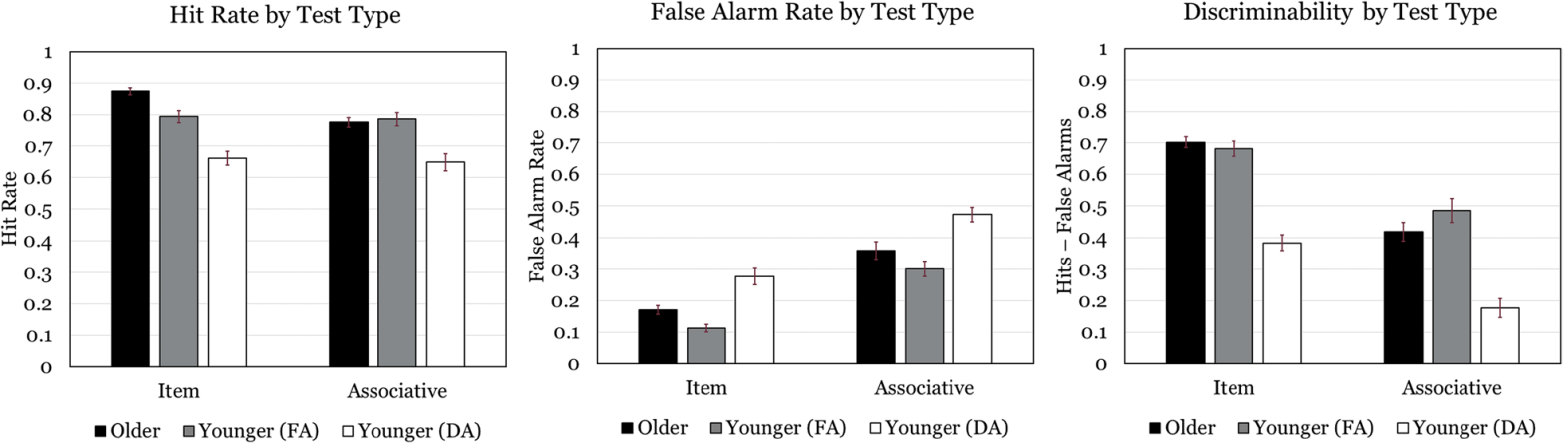
Hit rate, false alarm rate, and average discriminability for each test type and age/attention group. Although planned analyses compared two age/attention groups at a time, all three groups are graphically presented together in these and remaining figures to avoid redundancy. FA = Full Attention, DA = Divided Attention

**Fig. 3:**
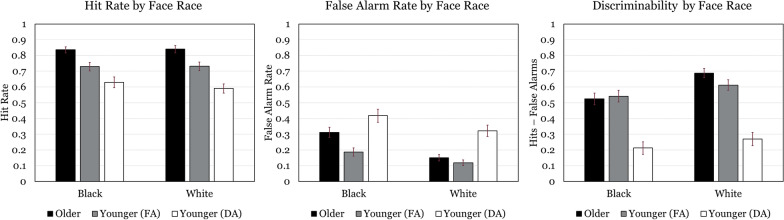
Hit rate, false alarm rate, and average discriminability for each face type and age/attention group. FA = Full Attention, DA = Divided Attention

**Fig. 4:**
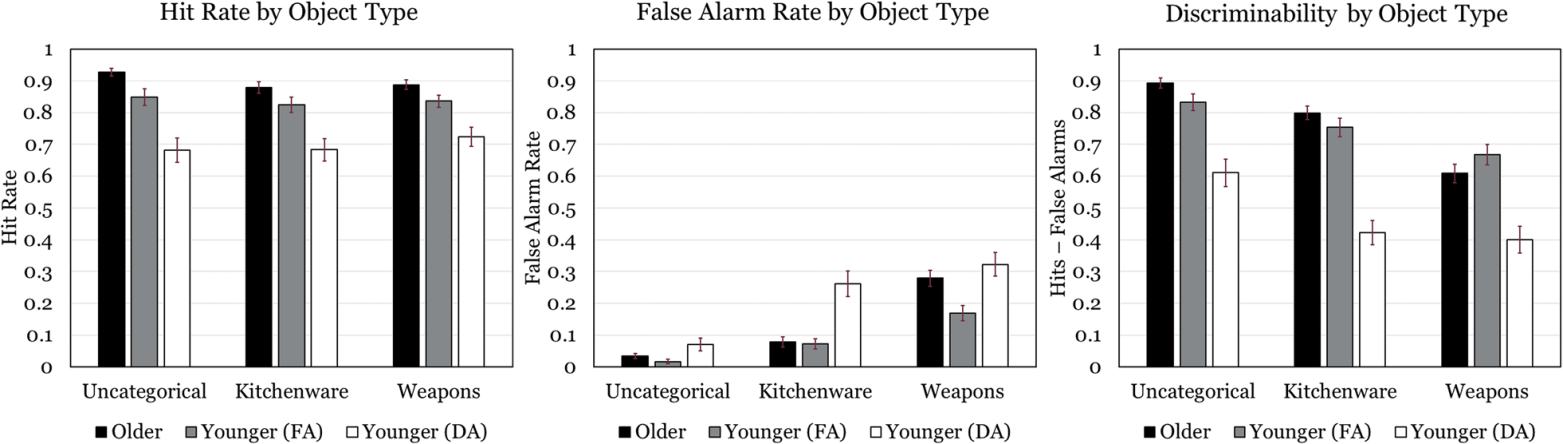
Hit rate, false alarm rate, and average discriminability for each object type and age/attention group. FA = Full Attention, DA = Divided Attention

**Fig. 5:**
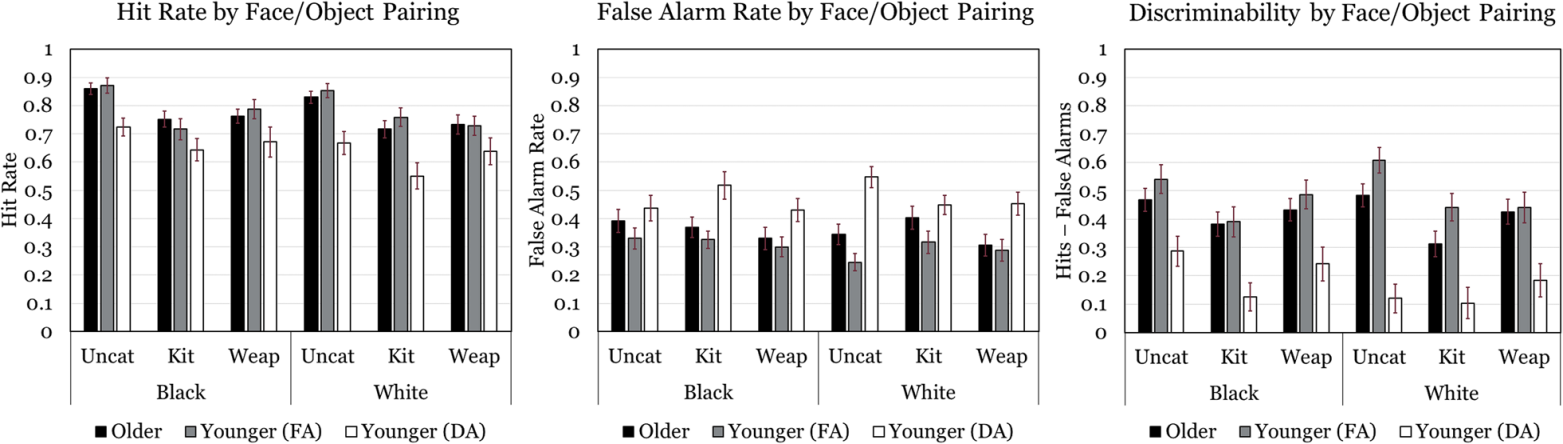
Hit rate, false alarm rate, and average discriminability for each associative memory combination in each age/attention group. FA = Full Attention, DA = Divided Attention

Our first set of analyses per each outcome and comparison investigated the effects and interactions of test type (item vs. associative) with age group (younger adults under full attention) so that we might detect an associative memory deficit affecting older adults. These analyses employed a 2 (age/attention group) × 2 (test type) mixed factorial design and are represented graphically in Fig. 2. The second and third sets of analyses investigated effects and interactions between item tests’ dependent measures (Black vs. White faces and uncategorical vs. kitchenware vs. weapon objects, respectively) with age/attention groups so that we might assess age/attention differences in memory for different items. Of particular interest here was the question of whether we detected own-race bias for White faces in either age group. The face race outcomes are represented in Fig. 3, and the object outcomes in Fig. 4. These analyses employed 2 (age/attention group) × 2 (face race) and 2 (age/attention group) × 3 (object type) mixed factorial designs. The final set of analyses per dependent measure and comparison explored differential associative memory for each face race and object type pairing to test our primary hypothesis that older adults would exhibit reduced associative deficit for Black/Weapon test pairs. These employed a 2 (Age/Attention group) × 3 (object type: uncategorical, kitchenware, and weapons) × 2 (face type: Black and White) mixed factorial design. Outcomes for these analyses are represented in Fig. 5.

### Full attention younger adults vs. older adults: discriminability

#### Main effects

No main effect of age was revealed. Discriminability for item memory (*M* = 0.69, *SD* = 0.13) was found to be significantly more accurate than associative memory (*M* = 0.45, *SD* = 0.22), *F*(1, 81) = 177.99, *p* < 0.001, *η*_*p*_^*2*^ = 0.69 (see Fig. 2). A main effect for face type was also significant, *F*(1, 81) = 19.46, *p* < 0.001, *η*_*p*_^*2*^ = 0.19, indicating that performance was higher for White faces (*M* = 0.65, *SD* = 0.20) compared to Black faces (*M* = 0.53, *SD* = 0.24) (see Fig. 3 for a graphical representation). A main effect of Object Type was also uncovered, *F*(2, 162) = 60.05, *p* < 0.001, *η*_*p*_^*2*^ = 0.43, such that uncategorical objects yielded greater discriminability (*M* = 0.86, *SD* = 0.14) than kitchenware (*M* = 0.78, *SD* = 0.16), *t*(82) = 4.48, *p* < 0.01, which in turn yielded greater discriminability than weapons (*M* = 0.64, *SD* = 0.20), *t*(60) = 6.37, *p* < 0.01 (see Fig. 4 for a graphical representation). No significant differences for memory among the unique face-object combinations within associative memory tests were detected (see Fig. 4).

#### Interactions

The central hypotheses of this experiment each aimed to address several multifaceted questions related to interactions among face type and object category on overall memory discriminability. In particular, we wanted to determine if the associative deficit hypothesis held for the present experiment. An age x test type interaction did manifest in support of this, *F*(1, 81) = 5.60, *p* < 0.02, *η*_*p*_^*2*^ = 0.07. Simple univariate ANOVAs split by age group revealed that this interaction stems from a larger associative memory deficit (performance on associative vs. item test trials) for older adults, *F*(1, 42) = 161.74, *p* < 0.001, *η*_*p*_^*2*^ = 0.79 than for younger adults, *F*(1, 39) = 47.09, *p* < 0.001, *η*_*p*_^*2*^ = 0.55. Thus, our data successfully replicate the overall associative deficit observed in previous experiments.

A significant age x object type was also detected, *F*(2, 162) = 4.88, *p* < 0.01, *η*_*p*_^*2*^ = 0.06. Simple univariate ANOVAs split by age group revealed that this interaction stems from a larger monotonic decrease in memory for uncategorical to kitchenware to weapons for older adults, *F*(2, 84) = 50.63, *p* < 0.001, *η*_*p*_^*2*^ = 0.55 than for younger adults, *F*(2, 78) = 15.48, *p* < 0.001, *η*_*p*_^*2*^ = 0.28. No further interactions were detected, including the central hypothesis that associative tests featuring Black faces and weapons would be more discriminable than other face/object pairings.

### Full attention younger adults vs. older adults: hits

#### Main effects

No main effect of age was revealed. Average hit rate for item memory (*M* = 0.84, *SD* = 0.10) was found to be significantly higher than for associative memory (*M* = 0.78, *SD* = 0.12), *F*(1, 81) = 24.02, *p* < 0.001, *η*_*p*_^*2*^ = 0.23 (see Fig. 2). No main effects of face race or object type were observed within item test analyses (see Figs. 3 and 4). The analysis of hit rates for associative memory tests revealed a main effect of object type, *F*(2, 162) = 23.74, *p* < 0.001, *η*_*p*_^*2*^ = 0.23, where pairs featuring uncategorical items (*M* = 0.85, *SD* = 0.15) produced more hits than kitchenware (*M* = 0.74, *SD* = 0.21) and weapons (*M* = 0.75, *SD* = 0.20), which were not different from one another (see Fig. 5).

#### Interactions

A significant test type x age interaction was observed, *F*(1, 81) = 17.01, *p* < 0.001, *η*_*p*_^*2*^ = 0.17. Follow-up simple effects tests of test type at each level of age revealed the interaction was driven by older adults producing more hits in the item test (*M* = 0.87, *SD* = 0.02) than the associative test (*M* = 0.78, *SD* = 0.02), *F*(1, 81) = 13.90, *p* < 0.001, *η*_*p*_^*2*^ = 0.15, whereas there was no age effect for associative memory tests. No further interactions were detected in any analysis of hits comparing these age groups.

### Full attention younger adults vs. older adults: false alarms

#### Main effects and interactions

No main effect of age on test type false alarm rate was revealed. Average false alarm rate for item memory (*M* = 0.33, *SD* = 0.17) was found to be significantly higher than associative memory false alarm rate (*M* = 0.14, *SD* = 0.09), *F*(1, 81) = 126.46, *p* < 0.001, *η*_*p*_^*2*^ = 0.61. A main effect of age on face item test false alarm rate was detected such that older adults (*M* = 0.23, *SD* = 0.17) produced more false alarms than younger adults (*M* = 0.15, *SD* = 0.14), *F*(1, 81) = 7.20, *p* = 0.009, *η*_*p*_^*2*^ = 0.08. This test also revealed a main effect of face race on face item test false alarm rates such that Black faces (*M* = 0.25, *SD* = 0.20) produced more false alarms than White faces (*M* = 0.14, *SD* = 0.12), *F*(1, 81) = 34.71, *p* < 0.001, *η*_*p*_^*2*^ = 0.30. A main effect of age on object item test false alarm rates was detected such that older adults (*M* = 0.13, *SD* = 0.11) produced more false alarms than younger adults (*M* = 0.09, *SD* = 0.10), *F*(1, 81) = 6.77, *p* = 0.01, *η*_*p*_^*2*^ = 0.08. This analysis also revealed a main effect of object type, *F*(2, 162) = 84.63, *p* < 0.001, *η*_*p*_^*2*^ = 0.51, where weapon tests (*M* = 0.23, *SD* = 0.17) produced more false alarms than kitchenware (*M* = 0.08, *SD* = 0.10), which in turn produced more false alarms than (*M* = 0.03, *SD* = 0.05). No main effects of age or race/object pairing on false alarms were found within the associative memory analyses.

A significant age × face race interaction was observed, *F*(1, 81) = 5.64, *p* = 0.02, *η*_*p*_^*2*^ = 0.07. Follow-up simple effects tests revealed older adults produced more false alarms for Black faces (*M* = 0.31, *SD* = 0.03) than for White faces (*M* = 0.15, *SD* = 0.02), *F*(1, 81) = 8.91, *p* = 0.004, *η*_*p*_^*2*^ = 0.10, whereas face race did not significantly affect younger adults’ hit rates. A significant age x object type interaction was found, *F*(2, 162) = 6.38, *p* = 0.002, *η*_*p*_^*2*^ = 0.07. Follow-up simple effects tests of age at each level of object type revealed this interaction was driven by older adults producing more false alarms for weapons (*M* = 0.28, *SD* = 0.03) than younger adults (*M* = 0.17, *SD* = 0.03), *F*(1, 81) = 9.64, *p* = 0.003, *η*_*p*_^*2*^ = 0.11, with no effects of age detected for the other object types. No further interactions were detected in any analysis of false alarms.

### Divided attention younger adults vs. older adults: discriminability

#### Main effects

Older adults (*M* = 0.56, *SD* = 0.15) outperformed younger adults under divided attention (*M* = 0.28, *SD* = 0.15) in both test types, *F*(1, 70) = 74.05, *p* < 0.001, *η*_*p*_^*2*^ = 0.51. Discriminability for item memory (*M* = 0.57, *SD* = 0.20) was significantly more accurate than associative memory (*M* = 0.32, *SD* = 0.22), *F*(1, 70) = 186.38, *p* < 0.001, *η*_*p*_^*2*^ = 0.73 (see Fig. 2). A main effect for face type was also significant, *F*(1, 70) = 13.42, *p* < 0.001, *η*_*p*_^*2*^ = 0.16, indicating that performance was higher for White faces (*M* = 0.52, *SD* = 0.29) compared to Black faces (*M* = 0.40, *SD* = 0.28) (see Fig. 3). A main effect of Object Type was also uncovered, *F*(2, 140) = 43.87, *p* < 0.001, *η*_*p*_^*2*^ = 0.39, such that uncategorical objects yielded greater discriminability (*M* = 0.78, *SD* = 0.22) than kitchenware (*M* = 0.65, *SD* = 0.25), *t*(71) = 5.05, *p* < 0.01, which in turn yielded greater discriminability than weapons (*M* = 0.53, *SD* = 0.23), *t*(71) = 4.34, *p* < 0.01. No significant differences among the face-object combinations within associative memory tests were detected (see Fig. 4).

#### Interactions

An age x memory test interaction manifested, *F*(1, 70) = 4.83, *p* = 0.03, *η*_*p*_^*2*^ = 0.07. Simple univariate ANOVAs split by age group revealed that this interaction stems from a larger associative memory deficit for older adults, *F*(1, 42) = 161.74, *p* < 0.001, *η*_*p*_^*2*^ = 0.79 than for younger adults under divided attention, *F*(1, 28) = 52.11, *p* < 0.001, *η*_*p*_^*2*^ = 0.65.

The age x object type analysis revealed an interaction between these factors, *F*(2, 140) = 5.12, *p* < 0.01, *η*_*p*_^*2*^ = 0.07. Simple univariate ANOVAs split by age group revealed that this interaction stems from a larger monotonic decrease in memory for uncategorical to kitchenware to weapons for older adults, *F*(2, 84) = 50.63, *p* < 0.001, *η*_*p*_^*2*^ = 0.55 than for younger adults under divided attention, *F*(2, 56) = 11.42, *p* < 0.001, *η*_*p*_^*2*^ = 0.29. No further interactions were detected. Overall, then, younger adults studying under divided attention performed worse than older adults, replicating findings elsewhere supporting the contention that age-related associative deficits do no a global reduction in representation density (cf., Benjamin, 2010).

### Divided attention younger adults vs. older adults: hits

#### Main effects

The test type x age analysis revealed no main effect of age, but did reveal a main effect of test type on hit rates such that item tests (*M* = 0.79, *SD* = 0.14) produced higher hit rates than associative tests (*M* = 0.73, *SD* = 0.13), *F*(1, 70) = 14.33, *p* < 0.001, *η*_*p*_^*2*^ = 0.17. The face race x age analysis revealed a main effect of age group such that older adults (*M* = 0.84, *SD* = 0.14) produced more hits than younger adults studying under divided attention (*M* = 0.61, *SD* = 0.17), *F*(1, 70) = 56.06, *p* < 0.001, *η*_*p*_^*2*^ = 0.45, whereas this analysis revealed no main effect of face race. The object type x age analysis revealed a main effect of age group such that older adults produced more hits (*M* = 0.90, *SD* = 0.10) than younger adults studying under divided attention (*M* = 0.69, *SD* = 0.18), *F*(1, 70) = 70.79, *p* < 0.001, *η*_*p*_^*2*^ = 0.50. No effect of object type on item test hit rates was revealed by this analysis. The age x face race x object type analysis of hit rates in associative memory tests revealed a main effect of age such that older adults (*M* = 0.78, *SD* = 0.17) produced more hits than younger adults studying under divided attention (*M* = 0.65, *SD* = 0.23), *F*(1, 70) = 19.36, *p* < 0.001, *η*_*p*_^*2*^ = 0.22. This analysis also detected a greater hit rate for Black faces (*M* = 0.75, *SD* = 0.20) than White faces (*M* = 0.70, *SD* = 0.22), *F*(1, 70) = 6.75, *p* = 0.01, *η*_*p*_^*2*^ = 0.09. Finally, this analysis detected a main effect of object type, *F*(2, 140) = 10.98, *p* < 0.001, *η*_*p*_^*2*^ = 0.14, where uncategorical objects produced the greatest hit rate (*M* = 0.78, *SD* = 0.18), followed by weapons (*M* = 0.71, *SD* = 0.23) followed by kitchenware (*M* = 0.68, *SD* = 0.22).

#### Interactions

The test type x age analysis revealed a test type x age interaction, *F*(1, 70) = 8.53, *p* = 0.005, *η*_*p*_^*2*^ = 0.11. Simple effects tests of age at each level of test type revealed the interaction was driven by older adults producing a greater effect of test type on hit rates, *F*(1, 70) = 90.45, *p* < 0.001, *η*_*p*_^*2*^ = 0.56, than younger adults, *F*(1, 70) = 19.36, *p* < 0.001, *η*_*p*_^*2*^ = 0.23. No further interactions were detected in any analyses.

### Divided attention younger adults vs. older adults: false alarms

#### Main effects

The test type x age analysis of false alarm rates revealed a main effect of age, *F*(1, 70) = 14.48, *p* < 0.001, *η*_*p*_^*2*^ = 0.17, where older adults (*M* = 0.26, *SD* = 0.14) produced fewer false alarms than younger adults studying under divided attention (*M* = 0.38, *SD* = 0.13). This analysis also yielded a main effect of test type on false alarm rates, *F*(1, 70) = 120.57, *p* < 0.001, *η*_*p*_^*2*^ = 0.63, such that item tests (*M* = 0.21, *SD* = 0.13) produced fewer false alarms than associative tests (*M* = 0.40, *SD* = 0.17). The face race x age analysis revealed a main effect of age group such that older adults (*M* = 0.26, *SD* = 0.14) produced more hits than younger adults studying under divided attention (*M* = 0.38, *SD* = 0.13), *F*(1, 70) = 12.52, *p* = 0.001, *η*_*p*_^*2*^ = 0.15. This analysis also revealed a main effect of face race such that Black faces (*M* = 0.35, *SD* = 0.22) produced more false alarms than White faces (*M* = 0.22, *SD* = 0.18), *F*(1, 70) = 31.00, *p* < 0.001, *η*_*p*_^*2*^ = 0.31. The object type x age analysis revealed an effect of age on false alarm rates, *F*(1, 70) = 11.33, *p* = 0.001, *η*_*p*_^*2*^ = 0.14, such that older adults (*M* = 0.13, *SD* = 0.11) produced fewer false alarms than younger adults studying under divided attention (*M* = 0.22, *SD* = 0.18). This analysis also revealed an effect of object type, *F*(2, 140) = 71.70, *p* < 0.001, *η*_*p*_^*2*^ = 0.51, such that weapons (*M* = 0.30, *SD* = 0.18) produced more false alarms than kitchenware (*M* = 0.15, *SD* = 0.18), which in turn produced more false alarms than uncategorical objects (*M* = 0.05, *SD* = 0.08). The age x face race x object type analysis of false alarm rates in associative memory tests revealed a main effect of age such that older adults (*M* = 0.36, *SD* = 0.25) produced fewer false alarms than younger adults studying under divided attention (*M* = 0.47, *SD* = 0.22), *F*(1, 70) = 8.54, *p* = 0.005, *η*_*p*_^*2*^ = 0.11. No other main effects on false alarm rates were found in these analyses.

#### Interactions

The object type x age analysis revealed an interaction between these factors, *F*(2, 140) = 7.83, *p* = 0.001, *η*_*p*_^*2*^ = 0.10. Simple effects tests of age at each level of object type showed this interaction was driven by a main effect of age group for kitchenware, *F*(1, 70) = 22.82, *p* < 0.001, *η*_*p*_^*2*^ = 0.25, where older adults had a lower false alarm rate (*M* = 0.08, *SD* = 0.02) than younger adults studying under divided attention (*M* = 0.26, *SD* = 0.03), whereas the other two object types yielded no simple age effects. No further interactions were detected in any analyses of false alarms comparing these two groups.

### Full attention vs. divided attention within younger adults: discriminability

#### Main effects

Younger adults under full attention (*M* = 0.58, *SD* = 0.20) outperformed younger adults under divided attention (*M* = 0.28, *SD* = 0.15) across all test types, *F*(1, 67) = 58.38, *p* < 0.001, *η*_*p*_^*2*^ = 0.47. Discriminability for item memory (*M* = 0.56, *SD* = 0.21) was significantly more accurate than associative memory (*M* = 0.36, *SD* = 0.26), *F*(1, 67) = 94.02, *p* < 0.001, *η*_*p*_^*2*^ = 0.58. A main effect for face type was also significant, *F*(1, 67) = 6.34, *p* = 0.001, *η*_*p*_^*2*^ = 0.01, indicating that performance was higher for White faces (*M* = 0.47, *SD* = 0.28) compared to Black faces (*M* = 0.40, *SD* = 0.28). A main effect of Object Type was also uncovered, *F*(2, 134) = 25.65, *p* < 0.001, *η*_*p*_^*2*^ = 0.28, such that uncategorical objects yielded greater discriminability (*M* = 0.74, *SD* = 0.23) than kitchenware items (*M* = 0.61, *SD* = 0.25), *t*(68) = 4.74, *p* < 0.01, which in turn yielded greater discriminability than weapons (*M* = 0.56, *SD* = 0.25), *t*(68) = 2.12, *p* < 0.05. No significant effects of face race by object type pairing within associative memory tests were detected. In addition, attention did not interact with test type, face type, or face-object pairing. Overall, analyses comparing these outcomes in each attention group reiterate that overloading attentional resources in younger adults does not reflect the same type of memory deficits observed with normal cognitive aging.

### Full attention vs. divided attention within younger adults: hits

#### Main effects

An attention group x test type analysis of younger adults’ hit rates revealed that those studying under full attention (*M* = 0.79, *SD* = 0.13) produced more hits than those studying under divided attention (*M* = 0.66, SD = 0.13), *F*(1, 67) = 22.64, *p* < 0.001, *η*_*p*_^*2*^ = 0.25. No effect of test type was found. An attention group x face race analysis of younger adults’ hit rates revealed younger adults studying under full attention (*M* = 0.73, *SD* = 0.17) produced more hits than those studying under divided attention (*M* = 0.61, *SD* = 0.17), *F*(1, 67) = 10.25, *p* = 0.002,, *η*_*p*_^*2*^ = 0.13. The attention group x object type analysis of younger adults’ hit rates revealed those studying under full attention (*M* = 0.84, *SD* = 0.15) produced more hits than those studying under divided attention (*M* = 0.70, *SD* = 0.18), *F*(1, 67) = 20.82, *p* < 0.001,, *η*_*p*_^*2*^ = 0.24. No effect of object type was revealed. The analysis comparing hit rates on associative memory tests revealed a main effect of attention group such that younger adults studying under full attention (*M* = 0.79, *SD* = 0.20) produced more hits than those studying under divided attention (*M* = 0.65, *SD* = 0.23), *F*(1, 67) = 15.78, *p* < 0.001,, *η*_*p*_^*2*^ = 0.19. This analysis also revealed test pairs with Black faces (*M* = 0.74, *SD* = 0.22) produced more hits than pairs with White faces (*M* = 0.71, *SD* = 0.23), *F*(1, 67) = 4.08, *p* = 0.048, *η*_*p*_^*2*^ = 0.06. Finally, it revealed an effect of object type, *F*(2, 134) = 10.91, *p* < 0.001, *η*_*p*_^*2*^ = 0.14, such that uncategorical objects produced the most hits (*M* = 0.79, *SD* = 0.20), followed by weapons (*M* = 0.71, *SD* = 0.24), and kitchenware (*M* = 0.68, *SD* = 0.24).

#### Interactions

No interactions between attention group and other factors on hit rates were observed in any analyses of hits comparing younger adults in each attention group.

### Full attention vs. divided attention within younger adults: false alarms

#### Main effects

An attention group x test type analysis of younger adults’ false alarm rates revealed that younger adults studying under full attention (*M* = 0.21, *SD* = 0.11) produced fewer false alarms than those studying under divided attention (*M* = 0.38, *SD* = 0.13), *F*(1, 67) = 41.77, *p* < 0.001, *η*_*p*_^*2*^ = 0.38. This analysis also revealed that the associative memory test (*M* = 0.37, *SD* = 0.16) produced more false alarms than the item memory test (*M* = 0.18, *SD* = 0.14), *F*(1, 67) = 142.66, *p* < 0.001, *η*_*p*_^*2*^ = 0.68. The attention group x face race analysis on false alarm rates also revealed those studying under full attention (*M* = 0.15, *SD* = 0.14) produced fewer false alarms than those studying under divided attention (*M* = 0.37, *SD* = 0.21), *F*(1, 67) = 34.82, *p* < 0.001, *η*_*p*_^*2*^ = 0.34. This analysis also revealed more false alarms for Black faces (*M* = 0.28, *SD* = 0.22) than for White faces (*M* = 0.20, *SD* = 0.18), *F*(1, 67) = 16.21, *p* < 0.001, *η*_*p*_^*2*^ = 0.20. The attention group x object type analysis revealed those studying under divided attention (*M* = 0.22, *SD* = 0.18) produced more false alarms than those studying under full attention (*M* = 0.09, *SD* = 0.10), *F*(1, 67) = 26.77, *p* < 0.001, *η*_*p*_^*2*^ = 0.29. This analysis also revealed a main effect of object type, *F*(2, 134) = 46.15, *p* < 0.001, *η*_*p*_^*2*^ = 0.41, such that weapons (*M* = 0.23, *SD* = 0.19) produced the most false alarms, followed by kitchenware (*M* = 0.15, *SD* = 0.19) and uncategorical objects (*M* = 0.04, *SD* = 0.08). The analysis comparing false alarm rates on associative memory tests revealed a main effect of attention group such that younger adults studying under full attention (*M* = 0.30, *SD* = 0.22) produced fewer false alarms than those studying under divided attention (*M* = 0.47, *SD* = 0.22), *F*(1, 67) = 25.04, *p* < 0.001,, *η*_*p*_^*2*^ = 0.27. No further main effects on false alarms were observed in any analyses.

#### Interactions

The attention group x object type analysis revealed an attention group x object type interaction, *F*(2, 134) = 5.46, *p* = 0.005, *η*_*p*_^*2*^ = 0.08. Simple effects tests of attention group at each level of object type revealed, significant effects of attention at each level of object. The strongest effect saw those studying under divided attention produced more false alarms for kitchenware (*M* = 0.26, *SD* = 0.03) than did those studying under full attention (*M* = 0.07, *SD* = 0.03), *F*(1, 67) = 23.26, *p* < 0.001, *η*_*p*_^*2*^ = 0.26, whereas this effect was weaker for weapons, *F*(1, 67) = 12.73, *p* = 0.001, *η*_*p*_^*2*^ = 0.16, and uncategorical objects, *F*(1, 67) = 7.91, *p* = 0.006, *η*_*p*_^*2*^ = 0.11.

The analysis comparing false alarm rates on associative memory tests revealed a significant attention group x object type x face race interaction, *F*(2, 134) = 4.27, *p* = 0.02, *η*_*p*_^*2*^ = 0.06. Figure 5’s visualization of false alarm rates indicates similar effects of age at each face/object type pairing, but the widest gap between full and younger attention younger adults appearing in the White/Uncategorical pairing, with the narrowest difference appearing in the Black/Uncategorical pairing.

### IAT scores and associative memory performance

Next, we conducted analyses to assess differences between the age groups in implicit bias toward black faces and also whether there were relationships within each age group between associative memory discriminability and bias revealed by IAT scores. IAT scores were calculated using the improved scoring algorithm (Greenwald et al., 2003), which also provides guidelines for discarding data if participants respond too quickly (< 300 ms) or too slowly (> 10,000 ms). Final scores on the IAT range from -2 to + 2, with negative scores indicating relative preference for White faces and positive scores indicating relative preference for Black faces. Cutoff absolute scores are 0.15 to 0.35 for slight bias, 0.35 to 0.65 for moderate bias, and beyond 0.65 for strong bias, and scores between -0.15 and 0.15 indicate no bias.

#### IAT mean differences

Generally, participants were biased in favor of associating White faces with “good” words at the expense of associating Black faces with “good” words. Older adults (*M* = -0.56, *SD* = 0.38) and younger adults under divided attention (*M* = -0.57, *SD* = 0.39) exhibited moderate bias and younger adults under full attention (*M* = -0.28, *SD* = 0.41) exhibited a light bias. The differences among the three subject groups were statistically significant, *F*(2, 90) = 5.22, *p* = 0.007, *η*_*p*_^*2*^ = 0.106, such that younger adults who studied under full attention during the memory task scored higher (less positive toward White faces) compared to the divided attention (*p* = 0.006) and older adult (*p* = 0.007) groups, which were not different from one another, and which both seemed to be more positive toward White faces and less so toward Black faces.

#### IAT covariation with associative memory

To examine the relationship between underlying implicit associations and explicit associative memory, we conducted analyses of our data with a series of linear mixed models using restricted maximum likelihood to avoid bias in parameter estimates. The first two models analyzed data obtained from older adults, and the second two models analyzed data obtained from younger adults.

The first model was specified to predict older adults' hits in associative memory tests from the fixed effects of face race (White vs. Black) and object (Uncategorized vs. Kitchenware vs. Weapon) with IAT entered as a covariate, all two-way interactions and the three-way interaction among face race, object type, and IAT score. It also examined the repeated effects of face race and object using an unstructured covariance structure nested within subjects. This model found no effects or interactions.

The second model was specified to predict older adults' false alarm rates in associative memory tests, but was otherwise designed in the same manner as the analysis of hits. This model found that IAT score negatively predicted accuracy for associative memory test trials featuring Black faces but not White faces, *b* = − 0.237, *SE* = 0.12, *p* = 0.048. In other words, older adults showing a preference for White faces on the IAT produced more false alarms for test image pairs featuring Black faces regardless of object type.

The third model was specified to predict younger adults' hits in associative memory tests from the fixed effects of face race, object, and attention at study (Full vs. Divided) with IAT entered as a covariate, all two-way interactions and the three-way interaction among face race, object type, and IAT score. It also examined the repeated effects of face race and object using an unstructured covariance structure nested within subjects. IAT scores did not predict accuracy in this model.

The fourth and final model was specified to predict younger adults' false alarm rates in associative memory tests and was designed in the same manner as the analysis of hits. This model found that IAT score negatively predicted accuracy for associative memory test trials featuring Black faces paired with uncategorized objects only among younger adults whose attention was divided during the study phase of the memory task, *b* = − 0.446, *SE* = 0.22, *p* = 0.048. In other words, younger adults in the divided attention group showing a preference for White faces on the IAT committed more false alarms for image pairs featuring Black faces and uncategorized objects.

## Discussion

The current study replicated the typical age-related associative memory deficit found in the extant literature extended to a new, socially relevant class of stimuli. It aimed more specifically to examine whether threatening objects (i.e., handheld weapons) are more likely to be associated in memory with Black faces than with White faces and whether this is the case in both younger and older adults as well as in younger adults under divided attention. Our major prediction was that age-related associative memory deficits in older adults would decrease when face-object pairs consisted of Black faces paired with weapons rather than paired with a non-threatening object. We predicted this decrease might occur due to cultural associations between Black faces and weapons. This association was thought to be especially strong among older adults due to a lifetime of exposure to cultural and media depictions of Black Americans as aggressive or more likely to engage in criminal behavior. However, this hypothesis was not supported.

Interestingly, because the current study manipulated the social variable of face race, our predominantly White sample (95% of older adults and 81% of younger adults) produced an own-race bias in face recognition, where both age groups better remembered White faces than Black ones, and this difference was somewhat greater among older adults. This replicates a stable effect in the social cognition of face memory (Rhodes & Anastasi, 2012). The current study was not designed to explore possible mechanisms responsible for this bias, and the sample was not racially equitable enough to reveal the symmetrical distribution of the effect often found between White and Black participants.

Finally, although our data did not yield any aggregate interactions between face race and object type pairings in the manner we hypothesized, our inclusion of the Face Races variant of the Implicit Association Test (Greenwald et al., 1998) allowed us to explore potential patterns and relationships between bias scores on this test and explicit associative memory accuracy. These analyses produced evidence that all three groups, but especially the older adults and the younger adults studying under divided attention, were biased in favor of associating White faces with “good” words at the expense of associating Black faces with “good” words (i.e., a general bias against Black faces). Furthermore, within the older adults group, race IAT scores correlated with false alarms for Black face-object associations, such that older adults with more implicit preference for White faces produced more false alarms associating Black faces with objects, reflecting a larger age-related associative memory deficit for Black than for white faces in older adults.

### Implications for cognitive aging

The data here contribute to the literature supporting the associative memory deficit that accompanies normal adult aging (Naveh-Benjamin, 2000). This pattern did not reflect a mere decline in attentional resources among older adults as the reason for this deficit, since younger adults under depleted attentional resources exhibited the poorest overall discriminability. Moreover, they produced the fewest hits and the most false alarms, whereas older adults tended merely to produce more false alarms without a similar decline in hits. Such results are in line with suggestions that the associative-binding deficit of older adults is a fundamental basic mechanism that is affected by age and is at least partially separate and independent from effects on cognition of a decline in attentional resources (e.g., Kilb & Naveh-Benjamin, 2007; Naveh-Benjamin & Mayr, 2018). It is worth noting that compared to previous literature on the associative deficit, the older adults in our sample exhibited relatively accurate memories. This may be due to the study duration (7 s per event), which is longer relative to previous literature using 4-5 s study durations. We decided on 7 s for the current experiment after a pilot test with older adults revealed that they performed near floor after studying for 5 s per event.^3^ Additionally, in contrast to the results for young under divided attention for the explicit memory measures, which do not seem to mimic the older adults ones, those obtained using mixed linear analyses incorporating IAT scores show some similarity between the results for older adults and those obtained in the group of younger adults that studied the information under divided attention. This provides some support to the suggestion that older adults’ decline in memory performance could at least partially be due to a decline in attentional resources (Craik, 1983, 1986).

However, our manipulation of face race and object type failed to yield more accurate associative memory among older adults when tested for Black faces paired with weapons, which we hypothesized would be schematically connected. Although this result does not replicate previous findings where related components at study were better remembered as studied pairs than unrelated components (e.g., Mohanty et al., 2016), there are important differences between the current experiment and those conducted previously that may explain the failure to find this interaction.^4^ First, stimuli that have elicited this interaction in the Mohanty et al. studies were not photographs as in the current study but words and simple graphical art images. Words and simple iconic images may produce more diffuse signals within semantic networks because they represent concepts directly, whereas photographs with more complex detail prime structural information before semantic information (e.g., Caramazza et al., 1990). Second, the facial stimuli from the current study were sourced from the MORPH database (Albert & Ricanek, 2008) which was assembled from publicly available mugshot photographs. We selected images from MORPH based on the people within them making an upright pose, neutral expression, and visual distinctiveness as assessed by the first and second authors. However, we may have selected images that were too discriminable, which may have bolstered older adults’ memories overall. The unamicable context of the photography (presumably taking place after arrest by police) may also be responsible for the failure to elicit a unique Black/Negativity association because all faces exuded a subtly grim visage. Additional study using a variety of controlled and normed facial stimuli may produce greater associative strength. Third, although earlier studies have demonstrated a general association between Black men and aggression in many perceptual and cognitive paradigms, the specific connection between Black men and weapons may not hold the preexisting paired associate strength as the stimuli used in previous studies. Finally, the presence and strength of racialized semantic associations may be highly variable among individuals due to idiosyncratic experiences, beliefs, and cultural knowledge, which is why we included the implicit association test results as a covariate in our linear models. However, we hesitate to make firm conclusions based on this final speculation, as we did not survey older adults about their social attitudes or personal experiences interacting with African Americans.

### Implications for social cognition, prejudice, and systemic racism

The current experiment studied phenomena that were hypothesized to be both consequences of and potential reinforcers of prejudice and systemic racism. Firstly, our manipulation of face race produced evidence of own-race bias of facial recognition among our predominantly White sample, and that this bias is driven by greater false alarms for Black test faces. Not itself directly predictive of racial animus, the prevailing explanations for the own-race bias are related to how individuals process facial categories: the perceptual learning explanation (Tanaka et al., 2004) and the categorization-individuation explanation (Hugenberg et al., 2010). Although our experiment was not designed to distinguish between these explanations, both are plausible given all of our subjects were sourced from university community in a rural Midwestern US town, which has a low percentage of Black residents and students.

An important finding from the current experiment was that poorer overall discriminability among older adults relative to younger adults was driven by a tendency to make false alarm errors. In an eyewitness memory scenario, this equates to erroneously identifying innocent suspects during perpetrator identification procedures. A meta-analysis of the extant research investigating older adults’ lineup identification accuracy revealed that they choose faces from lineups over twice as often as younger adults regardless of whether the perpetrator is present (Erickson et al., 2016). Our findings replicate this pattern within a basic memory paradigm and indicate that older adults, particularly those harboring negative bias for Black men (as reflected by the IAT scores), may confuse individuals not seen in threatening contexts for those who genuinely perpetrated crimes. Many real-world identifications are derived from show-ups—where police show a single photograph or a live suspect to a witness for identification—so recognition errors like those found here signify that innocent bystanders to crimes are imperiled by the intersection of cultural bias, faulty face recognition, and police procedures that do not adequately protect suspects.

Although our data did not yield aggregate interactions between face race and object type pairings in the manner we hypothesized, our inclusion of the Face Races variant of the Implicit Association Test (Greenwald et al., 1998) allowed us to explore potential relationships between bias scores on this test and explicit associative memory for Black faces paired with objects. These analyses produced evidence that race IAT scores correlated with associative memory for face-object associations regardless of object type pairing among older adults. Namely, the more bias older adults exhibited associating Black faces with “bad” words (perceiving them negatively), the greater were their false alarm rates in the associative memory tests featuring Black faces. That is, a tendency to incorrectly endorse a recombined image pair (a Black face and an object that appeared but not together during the study phase) as a pair originally presented together.

One question remaining is the reason for the patterns observed in older adults. Does it reflect negative animus toward Black Americans, which could be related to stronger unfavorable stereotypes in the older adults related to age per se (e.g., becoming more conservative with age), or to cohort effects resulting from stronger public endorsement of Black criminality stereotypes at the time when the older adults matured into adulthood, which have declined in recent decades (e.g., Kumah-Abiwu, 2020; Smiley & Fakunle, 2016)? Alternatively, these patterns may be due to the predominantly White sample of older adults having less experience interacting with Black Americans, reducing the opportunities to build positive associations (Bornstein, 1993).

To fully interpret these results, it is important to consider the range of IAT scores. As mentioned above, Greenwald et al. (2003) recommend cutoff scores for interpreting bias magnitude from 0 to |.15| for no bias, |.15| to |.35| for slight bias, |.35 to 0.65| for moderate bias, and beyond |.65| for strong bias. Positive scores indicate implicit bias associating Black faces and “good” words and negative scores indicate implicit bias of White faces with “good” words. Older adults committed more false alarms for pairs featuring Black faces as they went from relatively no bias to a strong bias associating White faces with “good” words. As always with the IAT, it is important to point out that this pattern could be due to negative animus toward Black Americans or due to more favorable attitudes toward White Americans. Older adults were recruited from a part of the USA that is predominantly White, meaning that older adults may simply have stronger associations with positivity for White individuals compared to Black individuals. Younger adults, in comparison, only committed more false alarms with image pairs featuring Black faces with uncategorized objects as they showed more favorable bias toward White faces. This unpredicted interaction may have been due to seemingly random nature of the uncategorical objects making pairs featuring them more difficult to discriminate, whereas schematically related objects (both weapons and kitchenware) were easier to remember due to these categories being repeatedly encoded throughout the experiment.

## Limitations and future directions

There are limitations in the current study. Particularly, the convenience sample of older adults consisted of predominantly White individuals and therefore did not provide enough data points to explore representative differences among participants’ races in the general population. Another possible limitation to the findings was the location where data were collected. The study took place on the campus of a large Midwestern US university. The cultural views of the participants toward social variables such as race and criminality may be unique in this versus other regions of the USA. Also, as previously stated, future directions would benefit from determining whether results here using MORPH faces generalize to other types of facial stimuli. More controlled stimulus photographs independently rated for various social and perceptual qualities (e.g., race prototypicality, masculinity, aggressiveness) would allow for systematic analysis of the relationship between faces and their associations. In addition, *less* controlled, candid photographs of individuals in the wild may enhance ecological validity. One final avenue for improving generalizability to real-world scenarios is to present objects and faces as integrated stimuli (i.e., where people actually hold the objects), rather than separate images side-by-side as in the current study.

## Conclusion

The current study explored evidence for age-related explicit associative memory deficits in a hitherto unexplored stimulus domain of different racial faces and object pairings. The results replicated and expanded the conditions under which older adults show an associative memory deficit—a deficit in binding together different components of an episode. In the current case, these components were a face and an object. Furthermore, although the results did not indicate any specific patterns with respect to the associative deficit and specific combinations of faces (i.e., black) and objects (weapons), they showed overall that the predominantly younger and older white participants in this study remembered better white over black faces. Finally, and importantly, using a measure of implicit racial bias (the Implicit Association Test), the current data indicate that older adults showed more bias than younger adults regarding black faces. In addition, within the older adults group, negative implicit attitudes toward Black faces were associated with higher false alarm rates in the face-object associative tests involving black faces. This and future studies aimed at replicating and expanding this research could contribute to future programs designed to provide strategies for weighing memory evidence throughout the justice system to combat racial biases and inequalities.

## Data Availability

Original data and syntax used for analyses are available to readers upon request.

## Abbreviations

ADH: Associative deficit hypothesis
IAT: Implicit association test

## Footnotes

1 In the case described, the car had backfired, resembling the sound of a gunshot, and the driver fled responding police for 20 min before pulling into a school parking lot where the shooting occurred.

2 Not all studies implicate a shooter bias, however. Correll et al. (2007b) compared trained police officers to a sample of college students and found the police did not exhibit such a bias against unarmed African Americans, and James et al. (2016) found that officers are less likely to shoot African Americans whether armed or unarmed.

3 A reviewer for an earlier draft of this manuscript brought up the possibility that the good memory of our older adult sample may have been due to the youth of some of our older adults, as three were under 65 years of age. We conducted an analysis comparing item and associative memory among younger adults under full attention and this truncated older adult sample. This analysis produced superficial differences from the reported analysis, but did not change patterns or significance.

4 Because null hypothesis statistical testing is not equipped to directly assess whether a null finding is "true", we used Faulkenberry's (2019) technique for using F-statistics, factor and error degrees of freedom, and the prior probability of the null hypothesis to calculate a Bayes Factor and posterior probabilities of the null and alternative hypotheses. Unlike NHST, Bayesian analysis does allow an estimation of the likelihood of the null hypothesis being true. To assess the likelihood that our null finding of associative memory for Black faces paired with weapons being equivalent to White faces paired with weapons, we first conducted a simple univariate analysis of variance investigating older adults' associative discriminably for White and Black faces paired with weapons. After revealing *F*(1, 42) = .025, we used these values and a prior probability of null set at .2 (based on the findings of Mohanty et al., (2016) to calculate *BF*_01_ = 6.47, meaning that the observed data are approximately 6.47 times more likely under the null hypothesis (posterior probability = .62) than under the alternative two-tailed hypothesis (posterior probability = .38).
